# Effect of apigenin on apoptosis induced by renal ischemia/reperfusion injury *in vivo* and *in vitro*

**DOI:** 10.1080/0886022X.2018.1497517

**Published:** 2018-10-03

**Authors:** Xiao Wang, Wei Wang, Jian-Zhong Wang, Cheng Yang, Chao-Zhao Liang

**Affiliations:** a Department of Urology, The First Affiliated Hospital of Anhui Medical University, Hefei, PR China;; b Institute of Urology, Anhui Medical University, Hefei, PR China;; c Department of Urology, Fuyang People’s Hospital, Fuyang, PR China

**Keywords:** Apigenin, renal ischemia/reperfusion (I/R) injury, apoptosis, Bcl-2, AKt

## Abstract

**Objectives:** This study aims to investigate the effects and molecular mechanisms of apigenin (ApI) on renal ischemia/reperfusion (I/R) injury *in vivo* and *in vitro*.

**Methods:***In vivo*, the left renal artery was clamped for 45 min and the right kidney was removed to study renal I/R injury on Sprague-Dawley (SD) rats. ApI was injected at 60 min before renal ischemia. *In vitro*, renal tubular epithelial cells (HK-2) were pretreated with or without ApI (20 uM) for 60 min and then treated with hypoxia/reoxygenation (H/R). Renal function, histology, cells apoptosis, and cell viability were tested. Furthermore, the potential molecular mechanisms were assessed.

**Results:** ApI pretreatment could significantly alleviated the renal function and the pathological damage as well as cells apoptosis after I/R injury. Meanwhile, ApI treatment protects H/R induced HK-2 cell apoptosis *in vitro*. The results of Western blot showed that ApI significantly increased the expressions of B-cell lymphoma 2 (Bcl-2) and phosphor-AKt (p-AKt), Phosphoinositide 3-kinase (PI3K), while down-regulated the expressions of Caspase3 and Bax induced by H/R injury.

**Conclusions:** ApI pretreatment can protect renal function against I/R injury and prevent renal tubular cells from apoptosis *in vivo* and *in vitro* which might through PI3K/Akt mediated mitochondria-dependent apoptosis signaling pathway.

## 1. Introduction

Acute kidney injury (AKI) is a major medical problem and catastrophic complication in hospitalized patients [1]. Renal ischemia/reperfusion (I/R) injury is a common cause of AKI. This injury initiates complex events within the kidney in renal injury and death of renal cells [2]. The molecular mechanisms of AKI remain poorly understood, and no effective therapeutic strategies to target AKI are available. Therefore, novel therapeutic solutions need to be explored to improve the outcomes of AKI.

The major pathological changes of AKI include damage of renal tubular epithelial cells, inflammation, endothelial cell injury and hemodynamic dysfunction [3]. Additionally, injury and death of tubular epithelial cells are recognized as the most crucial pathological changes among these characteristics in AKI. In ischemic AKI, tubular cells are lack of oxygen supply and may acquire injury caused by overproduced metabolic waste. The primary histological changes of renal tubular cells are characterized with sloughing of tubular epithelial cells, brush border loss, tubular lumen dilatation, and the formation of tubular casts caused by necrosis and apoptosis [4]. Rather than necrosis, apoptosis of renal tubular cells may be in charge of the dominant mode of injury. Therefore, the prevention of apoptosis of renal tubular cells is important to cure AKI [5–7]. Importantly, Phosphatidylinositol-3-kinase (PI3K)/Akt signal pathway regulates the renal repair after I/R injury [8–12]. A number of studies revealed that upregulation of phosphor-AKt (p-AKT) can ameliorate IR-induced renal/cerebral/heart injury by suppressing apoptosis [10,13,14]. Meanwhile, B-cell lymphoma 2 (Bcl-2) is involved in the mitochondria-mediating intrinsic apoptotic pathway [15] and can inhibit apoptosis in the renal I/R injury [16,17].

Apigenin (ApI) is a natural compound present in many fruits, and herbs, such as oranges, onions, and parsley [18]. ApI exhibits various biological roles as antiapoptosis, anti-inflammation, anticancer, antibacterial, and antioxidant effects [19–21]. ApI can also ameliorate I/R-induced injury in the heart, brain, and liver of rats and alleviate drug-induced nephrotoxicity in human renal proximal tubular epithelial cells *in vitro* [20,22,23]. This study aims to investigate the protective role of ApI against renal I/R injury in rats and whether the protective effects are induced through the PI3K/Akt pathway.

## 2. Materials and methods

### 2.1. Reagents

ApI (0.98%) was purchased from the National Institute for the Control of Pharmaceutical and Biological Products (Beijing, China) and dissolved in dimethyl sulfoxide (Beyotime Biotechnology, Jiangsu, China) with a final concentration of 0.01% in phosphate buffer solution (PBS). The rabbit polyclonal antibodies, PI3K, Bcl-2, Bax, Caspase-3, cleaved caspase-3 total AKt (t-AKT), and p-AKT antibodies, β-actin antibody, and secondary antibodies were obtained commercially from the Cell Signaling Technology (Beverly, MA). Cell Counting Kit-8 (CCK-8), terminal deoxynucleotidyl transferase-mediated deoxyuridine triphosphate-biotin nick end labeling (TUNEL) Apoptosis Assay Kit, and Annexin V/Propidium Iodide (PI) Apoptosis Detection Kit were acquired commercially from Beyotime Biotechnology (Jiangsu, China).

### 2.2. Animals model

Eighteen male Sprague-Dawley (SD) rats (200–250 g, 7–9 weeks old) were purchased from Shanghai Science Academy Animal Center (Shanghai, China). These rats were housed in a local facility for laboratory animal care and fed with a standard diet and water, according to local ethical guidelines. This study was approved by the Ethics Committee of Anhui Medical University. The rats were randomly divided into three groups with six rats in each group as follows: #I/R + saline group (IR), where the rats were subjected to intraperitoneal injection of normal saline for 1 h before renal ischemia; #I/R + ApI group (API), where the rats were administered with ApI (20 mg/kg, ip.) 1 h prior to I/R induction; and #sham-operated group (sham), where the rats were subjected to identical surgical procedure without occlusion of both renal pedicles. The dosage of ApI was determined according to previous studies [24]. Renal I/R injury was induced by a right nephrectomy and clamping of the left renal artery for 45 min [25,26]. The rats were anesthetized through an intraperitoneal injection of pentobarbital sodium (40 mg/kg body weight). After the median abdominal incision, first, the right kidney was removed first and then the left renal artery was clamped for 45 min with serrefine. After the clamp removal, adequate restoration of blood flow was assured before abdominal closure. Sham-operated animals underwent the same surgical procedure without clamping. Saline-treated animals received intraperitoneal injections of 0.9% sterile NaCl (1 mL) at 60 min before renal clamping. ApI-treated mice received intraperitoneal injections of ApI (20 mg/kg body weight) at 60 min before renal clamping. After the operation, the rats were kept in a warming blanket for 24 h with food and water available. The rats were sacrificed 24 h after the reperfusion. Their blood and kidneys were harvested for further analysis. Blood urea nitrogen (BUN) and serum creatinine (Scr) levels were assayed in the core laboratory of the First Affiliated Hospital of Anhui Medical University.

### 2.3. *In vitro* experiments

HK-2 cells were obtained from the Cell Bank of the Chinese Academy of Sciences (Shanghai, China). The cells were maintained in DMEM/F12 (1:1) (Gibco, Invitrogen, Carlsbad, CA) supplemented with 10% fetal bovine serum (Sijiqing Hangzhou, China) in 1:100 dilution of an antibiotic-antimycotic solution at 37 °7 in a 5% CO_2_ incubator. Exponentially growing cells were seeded in a culture dish at 1 × 10^5^ cells/mL in a complete medium for 24 h prior to the chemical treatment. HK-2 cells were randomly divided into four groups, as follows: (1) control group: the HK-2 cells were incubated with routine culture for an additional time of 27 h. (2) hypoxia/reoxygenation (H/R)-only (H/R) group: HK-2 cells were exposed to hypoxia for 24 h (5% CO_2_, 1% O_2_, and 94% N_2_) and reoxygenation for 3 h (5% CO_2_, 21% O_2_, and 74% N_2_). (3) H/R + ApI group: the HK-2 cells were incubated with ApI (20 µM) for 60 min prior to H/R initiation.

### 2.4. Histological analysis

Renal samples were fixed in formalin and embedded in paraffin. Renal sections were prepared and subjected to hematoxylin and eosin staining. Histopathological changes in kidney tissues were evaluated by a pathologist in a blinded fashion using a five-point quantitative scale according to the degree of tubular necrosis, tubular epithelial cell swelling, vacuolization, and desquamation as follows: 0, <10%; 1, 10–25%; 2, 25–50%; 3, 50–75%; and 4, 75–00% [27].

### 2.5. TUNEL staining

Paraffin-embedded kidney tissue sections were dewaxed and hydrated in graded ethanol, followed by permeabilization with 0.1 M sodium citrate, for 60 min at 60 °C. The apoptosis of renal tubular cells was examined using TUNEL staining. A one-step TUNEL Apoptosis Assay Kit was used according to the manufacturer’s instructions. The cells were observed under fluorescence microscopy for apoptosis. For quantification, 20 fields were randomly selected from each tissue section, and the number of TUNEL-positive cells per 1 mm^2^ was evaluated as previously described [28].

### 2.6. Cell viability assay

HK-2 cells were grown in 12-well plates at 1 × 10^5^/well and treated as described previously. Cell viability was measured using a CCK-8 assay following the manufacturer’s instructions. The percentage of cell proliferation was calculated using the following equation: (mean OD of treated cells/mean OD of control cells) × 100%.

### 2.7. Quantification of apoptosis

HK-2 cells were grown in 12-well plates and treated as described previously. Using Annexin V-FITC/PI staining quantified the apoptotic cells. By centrifugation and resuspension, we collected the cells in a 500-μL binding buffer. After that, the cells were stained with 5 μL PI and 5 μL Annexin V-fluorescein isothiocyanate by incubation for 5 min. The cells were analyzed using flow cytometry (Becton Dickinson FACS Vantage SE, San Jose, CA). Results were demonstrated as quadrant dot plots and each type of cells was described in percentages of the number of all stained cells.

### 2.8. Western blot analysis

HK-2 cells were treated as indicated and then washed twice with cold PBS, after which they are harvested. The whole cell extracts were lysed in a buffer containing complete protease inhibitor cocktail and then the lysates were centrifuged at 15 000×*g* for 30 min at 4 °C. The supernatants were collected for Western blot analysis. Equal amounts of cellular proteins were electrophoresed in 12% SDS-polyacrylamide gel electrophoresis, and then the proteins were transferred to polyvinylidene difluoride membranes (Millipore, Billerica, MA). The membranes were blocked with a blocking buffer (5% dried nonfat milk in Tris-buffered saline) for 1 h, and then the membranes incubated with specific primary antibodies overnight at 4 °C. After three washes with Tris-buffered saline, the blots were incubated with horseradish peroxidase-labeled-conjugated secondary antibodies for 2 h. The membranes were detected by enhanced chemiluminescence reagents (American Bioscience, Blauvelt, NY) and autoradiography. Band intensity was measured quantitatively and analyzed using the Quantity One software (Bio-Rad, Pleasanton, CA).

### 2.9. Statistical analysis

Experimental data are presented as means ± SD Differences between mean values were assessed using one-way ANOVA and Tukey’s multiple comparison tests using SPSS Software version 20.0 (SPSS Inc., Chicago, IL). Values at *p*<.05 were considered statistically significant.

## 3. Results

### 3.1. ApI protected renal function from renal I/R injury

**Figure 1.**
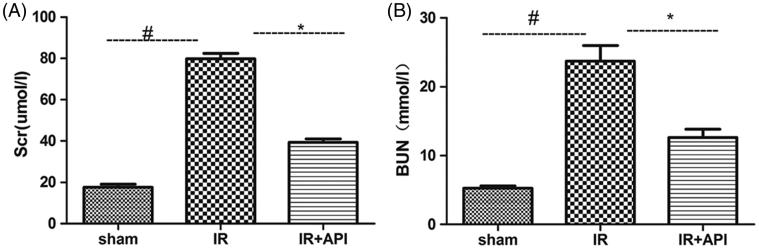
The Scr (A) and BUN (B) values measured after 45 min of ischemia followed by 24 h of reperfusion. #*p* < .05 vs. sham group, *p < .05 vs. IR group.

Rats in the IR group exhibited a 4.5-fold increase in the Scr level (78.8 ± 3.4 μmol/L) and approximately 4.5-fold increase in the BUN level (23.8 ± 3.7 mmol/L) compared with that in the sham group. These results indicated a significant deterioration of the renal function in the IR group (*p*<.005). The Scr (39.5 ± 6.4 μmol/L) and BUN levels (12.6 ± 1.2 mmol/L) were lower in the API group than that in the IR group (*p*<.05), which indicated that ApI can attenuate IR injury-induced renal dysfunction (Figure 1(A,B)).

### 3.2. ApI on histological changes after renal I/R injury

**Figure 2.**
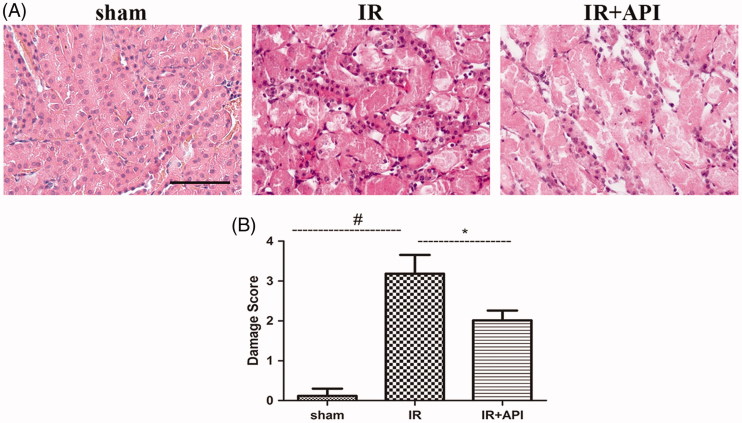
(A) Representative micrographs of H&E staining and (B) Quantitative damage score of H&E staining. #*p* < .05 vs. sham group, *p < .05 vs. IR group, Scale bar =100 µm.

Compared with the sham group, renal I/R induced significantly induced kidney injury characterized with morphological changes, such as brush border loss, tubular necrosis dilation, tubular epithelial cell swelling, vacuolization, and desquamation as shown (Figure 2(A)). However, ApI significantly ameliorated the IR-induced histological changes (*p*<.05), such as tissue injury, as assessed using a five-point scoring system. Quantitative scale evaluation showed that the damage scores of the IR, sham, and API groups were 3.12 ± 0.18, 0.15 ± 0.15, and 2.1 ± 0.17 points, respectively (Figure 2(B)).

### 3.3. ApI improved I/R induced renal proximal tubular epithelial cell apoptosis *in vivo*

**Figure 3.**
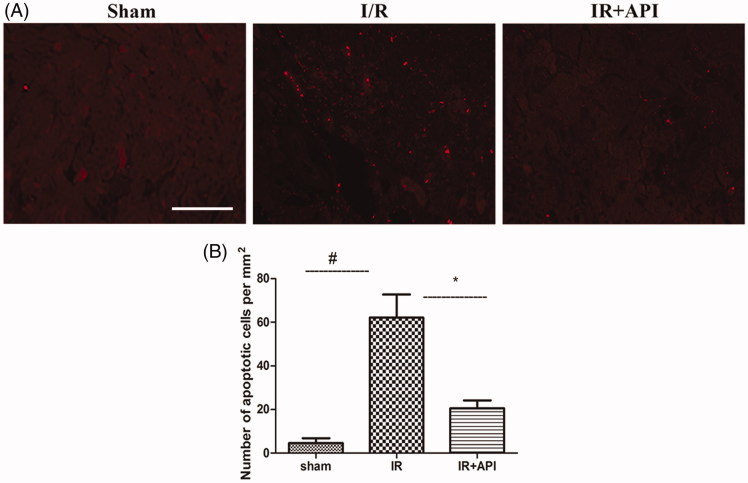
(A) Representative micrographs of Tunnel staining. (B) #*p* < .05 vs. sham group, *p < .05 vs. IR group, Scale bar =100 µm.

Renal proximal tubular epithelial cell apoptosis was determined with TUNEL staining in the sham, IR, and API groups. As shown in Figure 3(A), I/R injury induced an evident renal proximal tubular epithelial cell apoptosis (45.6 ± 4.8/mm^2^). Cell apoptosis was reduced notably in the API group (26.4 ± 4.3/mm^2^). Quantitative analysis showed that ApI pretreatment (API group) inhibited the I/R-induced increase in apoptosis cells (*p* < .05) (Figure 3(B)).

### 3.4. ApI blocked H/R induced renal tubular epithelial cells apoptosis *in vitro*

**Figure 4.**
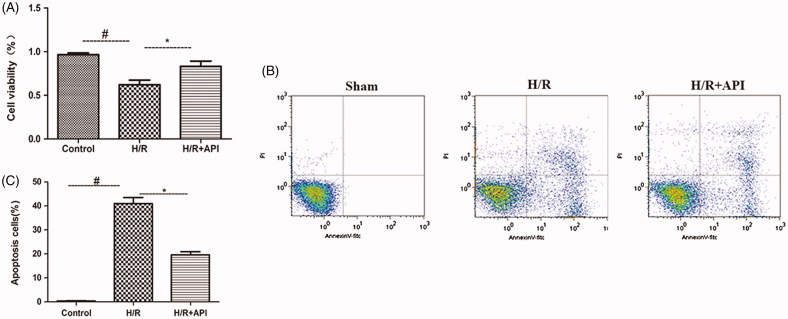
(A) CCK-8 method was employed to detect cell viability after the treatment. (B) Representative images of FACS to measure apoptosis of HK-2 cells after the treatment. (C) Quantitation of apoptotic cells in 10000 cells. #*p* < .05 vs. Control group, *p < .05 vs. H/R group.

Effects of ApI on the H/R-induced change in the cell viability of HK-2 cells were determined quantitatively with the CCK-8 assay. As shown in Figure 4(A), cell viability of HK2 cells was significantly decreased after treatment with H/R (*p* < .05). However, ApI pretreatment 60 min before the start of H/R significantly increased the cell viability in the API group (*p* < .05).

In addition, the effects of ApI on H/R-induced apoptosis were further tested through flow cytometric analysis in the HK-2 cells. As shown in Figure 4(B,C), HK-2 cell apoptosis rate increased significantly in the H/R group (0.41 ± 0.02) compared with that in the control group (*p* < .05). Pretreatment with ApI significantly reduced the H/R-induced cell apoptosis rate (0.19 ± 0.01) (*p* < .05).

### 3.5. Mechanisms of API preventing I/R-induced renal tubular cells apoptosis

**Figure 5.**
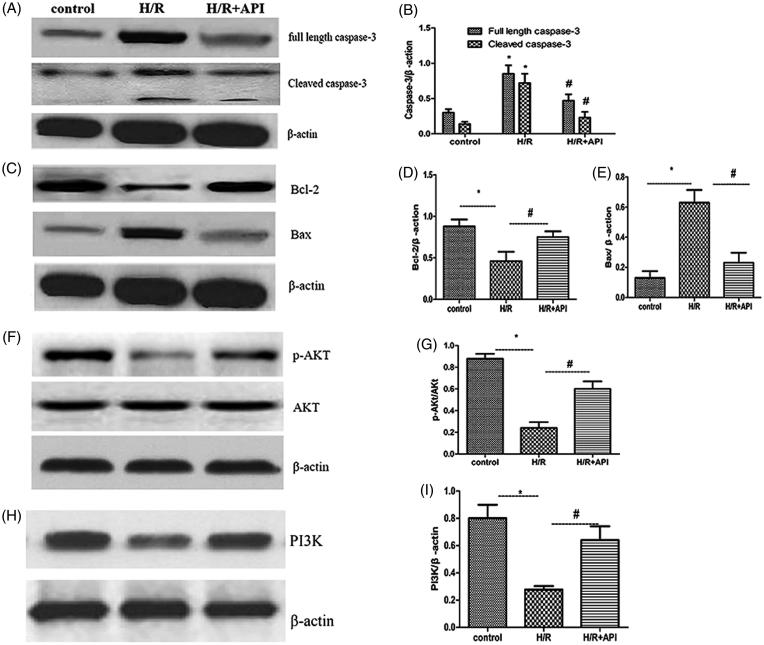
Western blot analysis of Caspase-3 and Cleaved-caspase3 (A), Bcl-2 and Bax (B), p-AKT and AKT (C). (B,D,E,F,G) and PI3K (H,I). Semi-quantitative analysis of western blot for different proteins. *p < .05 vs. Control group, #*p* < .05 vs. H/R group.

To investigate mechanisms of API that prevent I/R-induced renal tubular cells apoptosis, we detected the expressions of proteins participating in the mitochondria apoptotic signaling pathways. As shown in Figure 5(A,C,F,H), ApI significantly inhibited Caspase-3 activity in the HK-2 cells 3 h after H/R and decreased the expression of the cleaved Caspase-3 (*p* < .05). Additionally, Western blot assays of Bcl-2, Bax, proteins showed that ApI downregulated the expression of the proapoptotic protein Bax and upregulated the expression of the antiapoptotic protein Bcl-2, as shown in Figure 5(C,D) (*p* < .05, H/R group vs. H/R + API group). Furthermore, H/R significantly decreased PI3K and the p-AKT/AKT radio in the H/R group, and ApI upregulated the PI3Kand p-AKT/AKT expression radio in the API group, as shown in Figure 5(F– I) (*p* < .05, H/R group vs. API group).

## 4. Discussion

This study investigated the potential therapeutic effects and mechanisms of ApI on renal I/R injury. Biochemical tests revealed altered Scr and BUN levels. Histological analyses demonstrated significant pathological changes indicated by acute tubular necrosis, renal tubular expansion, renal tubular epithelial cell exfoliation, and proteinaceous cast development. ApI significantly attenuated the pathological changes associated with ameliorated renal function.

Renal I/R injury initiates interrelated sequence of events within the kidney, and these events result in renal injury and death of renal tubular cells. Endothelial dysfunction and tubular cell injury through the apoptotic pathway, ATP depletion, and proinflammatory cytokines have all been studied [29,30]. Given that apoptosis is associated with I/R injury in the kidney, we studied whether ApI ameliorated renal function through apoptosis in kidney I/R injury. In this study, renal tubular epithelial cell apoptosis was investigated in both in isolated rat kidneys and in HK-2 cells. TUNEL and flow cytometric analyses confirmed that I/R injury can cause renal tubular epithelial cell apoptosis. ApI pretreatment can significantly inhibit renal tubular epithelial cell apoptosis. In addition, ApI pretreatment can also reduce the activity of apoptosis-related Caspase-3 protein, downregulated full-length Caspase-3, and the cleaved Caspase-3 expression induced by the I/R. These results suggested that the nephroprotective effects of ApI against the renal I/R injury may be attributed to its inhibition of renal tubular epithelial cell apoptosis by suppressing the activity of Caspase-3. The Bcl-2 family members play an important role in the mitochondria-mediating intrinsic apoptotic pathway [16,31]. We observed that ApI pretreatment suppressed the expression of protein Bax and increased the protein Bcl-2 in rats. The Bax/Bcl-2 ratio significantly decreased by the precondition with ApI, which contributed to the sensitivity of cells to an apoptosis/death signal. This result suggested that the nephroprotective effects of ApI in the cultured HK-2 cells may be involved Bcl-2 signaling pathway.

PI3K/Akt pathway plays a critical role in regulating cell growth and survival [32]. Recently, this pathway has been implicated in the protection of liver, brain, heart, and kidney against I/R injury by moderating cell apoptosis [8,10,33,34]. We investigated the effect of ApI on AKT activation. H/R injury, ApI, and pretreatment exerted no influence on the expression of t-AKT. However, ApI administration can further increase the H/R-induced PI3K, and p-AKT expression. Thus, we concluded that PI3K/Akt signaling activation may suppress IR-induced apoptosis in the kidney.

## 5. Conclusions

In summary, this study showed that ApI exerted protective effects against the I/R-induced renal injury in rat kidneys and the cultured HK-2 cells. These nephroprotective effects were exerted through the Bcl-2 and PI3K/Akt signaling pathways, which blocked I/R-induced apoptosis. Nevertheless, the possible involvement of ApI-induced p-AKt activation could involve in additional pathways is unclear. Therefore, further studies should determine the pathways involved in p-Akt activation under renal IR injury.

## Limitations of this study

Possible involvement of ApI-induced p-AKt activation involve in additional pathways is unclear. Therefore, further studies should determine the pathways involved in p-Akt activation under renal IR injury.
